# Wavelet-Based Pattern ERG Biomarkers Outperform Temporal Amplitude Measures for Functional Stratification in Optic Nerve Disease

**DOI:** 10.1167/tvst.15.3.13

**Published:** 2026-03-11

**Authors:** Yousif J. Shwetar, Brett G. Jeffrey, Melissa A. Haendel

**Affiliations:** 1 Joint Department of Biomedical Engineering, University of North Carolina and North Carolina State University, Chapel Hill, NC, USA; 2 Ophthalmic Genetics and Visual Function Branch, National Eye Institute, National Institutes of Health, Bethesda, MD, USA; 3 Department of Genetics, University of North Carolina, Chapel Hill, NC, USA

**Keywords:** wavelet transform, pattern electroretinography, inherited retinal disease, inherited optic neuropathy, biomarker, signal processing

## Abstract

**Purpose:**

To extend wavelet analysis of pattern electroretinography (PERG) from macular cone to retinal ganglion cell (RGC) dysfunction in optic nerve disease (OND) by validating Symlet-2 (sym2) discrete wavelet transform (DWT) features.

**Methods:**

From the open access PERG–Institute of Applied Ophthalmobiology (IOBA) dataset, 58 recordings from OND subjects and 262 recordings from healthy volunteers (HVs) were analyzed. Five pre-selected sym2 coefficients (D5-2, D6-2, D6-3, A6-3, A6-4) were quantified. Their correlations with canonical amplitudes (|P50–N35|, |N95–P50|) and group separation (rank-biserial effect size, |*r_rb_*|) were analyzed. We also assessed a previously defined DWT energy index based on the Daubechies 8 mother wavelet (7N), capturing RGC activity.

**Results:**

The macular cone–specific sym2–D6-2 correlated tightly with |P50–N35| in HVs (*r_corr_* = 0.95) and OND subjects (*r_corr_* = 0.97). In contrast, sym2–A6-4 (112–150 ms, 0–13 Hz) was best suited to capture differences between the HV and OND groups (|*r_rb_*| = 0.549), compared to |N95–P50| (|*r_rb_*| = 0.358). Bootstrap benchmarking confirmed that sym2–A6-4 outperformed |P50–N35| and |N95–P50| (Δ|*r_rb_*| = 0.362 and 0.187; *P_boot_* = 0.005 and 0.036, respectively). The 7N feature failed to yield effective results on all measures (|*r_rb_*| = 0.084).

**Conclusions:**

Sym2 DWT features provide compartment-specific, multidimensional biomarkers that outperform traditional canonical peaks for both macular cone (sym2–D6-2) and RGC (sym2–A6-4) assessment. Future work should validate these biomarkers in a large, diverse, genetically and phenotypically characterized external cohort to confirm generalizability and clinical utility.

**Translational Relevance:**

Sym2 wavelet indices provide robust and sensitive PERG biomarkers that could serve as quantitative endpoints in clinical trials.

## Introduction

Electroretinography (ERG) is the gold standard for objective functional testing of the retina, translating light-evoked bioelectric activity into quantifiable waveforms that index the health of photoreceptors and downstream neurons.^1^^,^^2^ Within the ERG family, pattern ERG (PERG) is uniquely suited to capture both macular cone and retinal ganglion cell (RGC) function, as it isolates cone-driven postreceptoral responses under photopic, pattern-reversal stimulation.^3^ PERG waveform structure consists of a positive deflection at ∼50 ms (P50) predominantly driven by the macular cones, followed by a negative trough at ∼95 ms (N95) representing RGC activity. The PERG reflects visual processing in both proximal and distal retinal layers, thereby providing a biomarker for an array of eye diseases, including inherited retinal diseases (IRDs) and optic nerve disease (OND).^4^^–^^7^

IRDs encompass a genetically and phenotypically heterogeneous group of disorders that together affect ∼1 per 2000 to 3000 individuals worldwide.^8^^–^^10^ Among IRDs, those with predominant macular involvement (macular-predominant IRDs [mpIRDs]), such as Stargardt disease, exhibit early attenuation of the PERG.^11^^–^^13^ Alternatively, the N95 component of the PERG is degraded in OND, such as Leber's hereditary optic neuropathy and optic atrophy, as these conditions primarily affect RGCs.^14^^–^^16^

Though PERG has demonstrated clinical utility, analysis is confined to scalar measures such as peak amplitudes and implicit times.^3^^,^^17^ These metrics do not capture the rich, multidimensional responses embedded in the waveform. Discrete wavelet transform (DWT) offers a powerful alternative, as it decomposes PERG signals into time–frequency indices that preserve both temporal localization and spectral detail.^18^^,^^19^ Previous studies in ERG have successfully utilized DWT to extract robust biomarkers, underscoring their utility in diagnosis and monitoring clinical outcomes.^20^^–^^37^ Specifically, our previous work identified a PERG DWT index, sym2–D6-2, as a highly sensitive measure of macular cone activity, demonstrating improved discriminatory performance compared to traditional amplitude measures in mpIRDs (Table 1).^38^ Note that wavelet indices are denoted as a mother wavelet function and wavelet decomposition level index. For example, sym2–D6-2 is the second detail index at the sixth level of decomposition using the Symlet-2 mother wavelet (which has two vanishing moments).

Although those findings potentially identified a sensitive marker of macular cone response, they also laid the groundwork for an analogous exploration of RGC-centric features. In the current study, we therefore aimed to

1. Validate the previously identified macular cone marker (sym2–D6-2) in an independent OND cohort, ensuring continuity with our earlier findings.^38^
2. Identify and validate novel sym2 wavelet indices specifically sensitive to RGC activity, using a carefully selected OND cohort characterized by impaired N95 responses.
3. Develop complementary indices from the Haar wavelet, leveraging its superior temporal resolution and higher levels of decomposition (compared to other mother wavelets) to isolate transient components of the PERG.
4. Independently evaluate the previously proposed Daubechies 8–derived 7N coefficient, originally described in glaucoma, for its generalizability as an RGC marker in OND.

By establishing multidimensional PERG biomarkers sensitive to both macular cone and RGC dysfunction, we aimed to enhance diagnostic precision and identify sensitive endpoints for monitoring disease progression and therapeutic outcomes.

## Methods

### Data Source and Ethical Compliance

**Figure 1.**
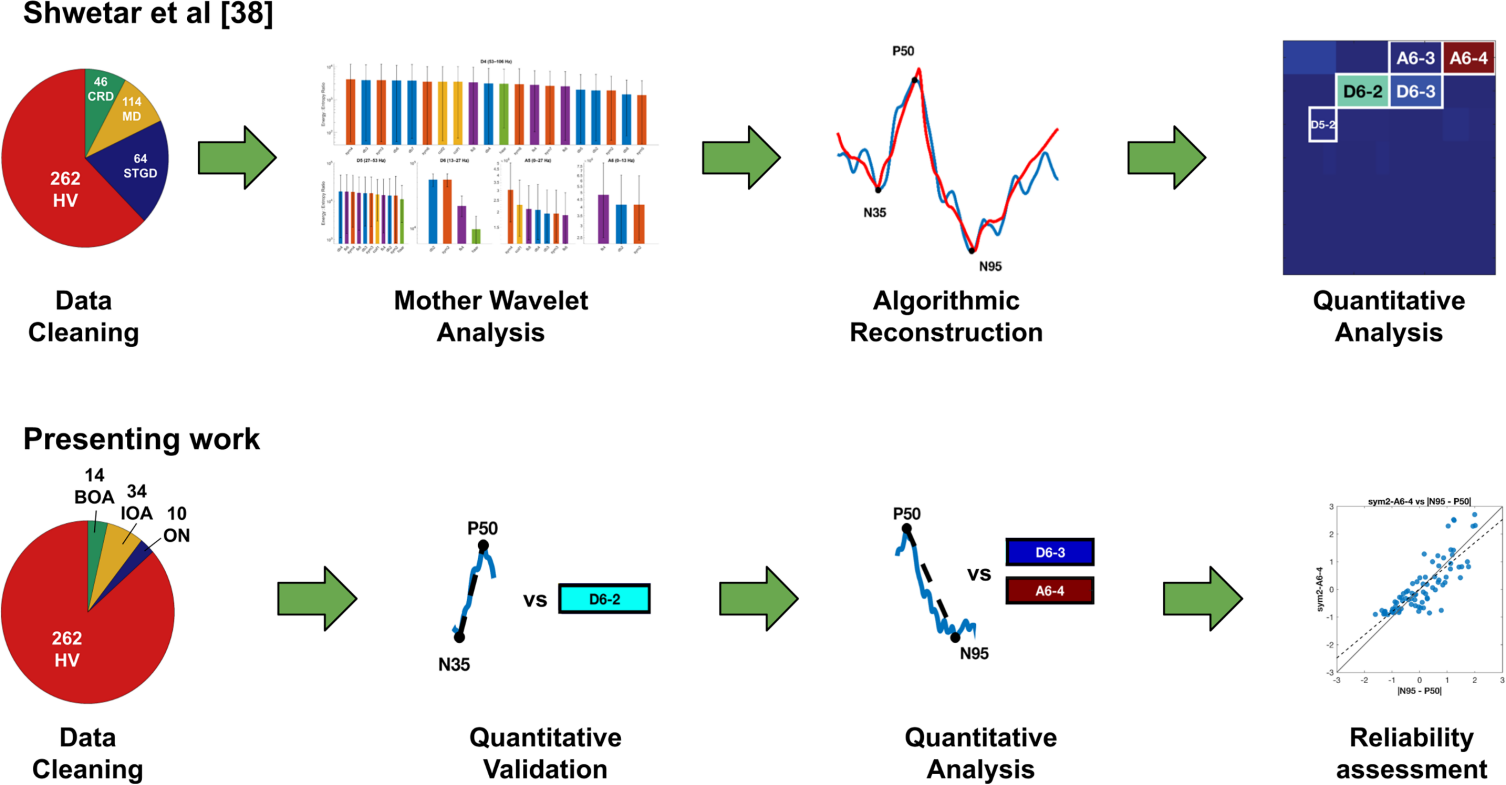
**Study roadmap and comparison with prior work.** (*Top*) Our previous study of macular cone activity^38^ obtained and cleaned the dataset (pie-chart of diagnostic categories and counts); screened 20 mother wavelets screened using the energy-to-entropy metric, identifying the highest ratio for sym2 at level D6; used algorithmic reconstruction to identify indices capturing canonical peaks (N35, P50, N95) using an algorithmic reconstruction approach; and quantified these indices, yielding Symlet-2 (sym2) indices D5-2, D6-2, D6-3, A6-3, and A6-4. (*Bottom*) The current study obtained a new OND cohort; validated the cone-driven feature sym2–D6-2; quantitatively evaluated RGC-dominant indices sym2–D6-3, sym2–A6-4, and mean(D6-3, A6-4); and assessed reliability using ICC(2,1). Additional work in the present study not included in the graphic above includes evaluation of effect sizes and the development of a complementary, phase-shift–free, time–frequency representation of the PERG using the Haar mother wavelet. CRD, cone–rod dystrophy; MD, macular dystrophy; STGD, Stargardt disease; BOA, bilateral optic atrophy; IOA, inherited optic atrophy; ON, optic neuropathy.

PERG data were obtained from the openly available PERG–Institute of Applied Ophthalmobiology (IOBA) repository hosted on PhysioNet.^39^^,^^40^ The archive is composed of 1354 PERG recordings obtained at the IOBA at the University of Valladolid (Valladolid, Spain) between 2003 and 2022. Data collection conformed to the tenets of the Declaration of Helsinki and received approval from the respective ethics committees. Informed consent was obtained from each subject. The current work herein is on fully de-identified traces and thus qualified as non-human subject research, requiring no additional review. Our overall methodological approach and its continuity with prior work are illustrated in Figure 1.

### Participant Selection and Clinical Groups

To systematically evaluate RGC function using DWT, we first identified a cohort representative of changes in RGC pathology. To do this, we retained all recordings labeled as “Inherited optic atrophy,” “Bilateral optic atrophy,” or “Optic neuropathy” in the PERG–IOBA metadata and collectively referred to this group as OND. Recordings labeled as “Normal” with no concomitant ocular diagnosis served as the healthy volunteer (HV) control group. There were 120 recordings of HV subjects with the text “Mercury poisoning” in the comment's column, which we excluded from analysis. Only recordings from a subject's first visit were retained. Traces lacking a visual acuity (VA) recording were removed from analysis. The final analytic sample contained 58 recordings from 15 OND subjects and 262 recordings from 67 HVs, with additional demographic information as shown in Table 2. All subjects had both left-eye (LE) and right-eye (RE) recordings, with some contributing multiple recordings per visit. Thus, recordings were averaged within visit and across eyes to yield a single subject-level measure, an approach appropriate for inherited bilateral conditions and necessary to avoid pseudoreplication.

### PERG Acquisition Parameters

Recordings were obtained using the MonPack 120 system (Metrovision, Pérenchies, France). All PERG recordings were elicited with a 12° × 16° black-and-white checkerboard reversing at 4 reversals per second (rps). Testing was performed under photopic conditions in a darkened room (∼4 lux) at a viewing distance of 1 meter with optical correction as needed; stimuli were presented on a cathode ray tube (CRT) monitor (75 Hz) with high contrast (∼100%) and white luminance > 80 cd/m^2^. Responses were recorded binocularly. Subjects were tested without dark adaptation or pharmacologic pupil dilation. Impedance was maintained below 5 kΩ (and background noise below 5 µV). Bioelectric activity was recorded with a gold-foil corneal electrode referenced to the ipsilateral outer canthus and grounded at the forehead. Signals were hardware bandpass filtered 1 to 100 Hz and digitized at 1700 Hz. For each eye, at least 100 artifact-free sweeps were averaged; sweeps exceeding ±8 µV in fast-rejection mode or ±50 µV in slow-rejection mode were automatically discarded and replaced by interpolation, in accordance with the 2012 International Society for Clinical Electrophysiology of Vision PERG standard.^41^

### Preprocessing

A 150-ms epoch centered on stimulus onset was exported for every recording. Each epoch was linearly detrended in MATLAB (MathWorks, Natick, MA) using the detrend function by fitting a 0th-order polynomial to the signal and subtracting it to remove DC offset. The final sample was duplicated once so the trace contained exactly 256 points (a power of two lengths required for dyadic decomposition). PERG metrics were identified on the average time-domain waveform for each eye using fixed latency windows. N35 was defined as the most negative deflection within 25 to 40 ms, P50 as the most positive deflection within 40 to 60 ms, and N95 as the most negative deflection within 85 to 115 ms relative to stimulus onset. Amplitudes were computed as |P50–N35| and |N95–P50|. All values and points were manually verified by the first author (YJS).

### Discrete Wavelet Transform

Wavelet-based time–frequency analysis can be implemented using either the continuous wavelet transform (CWT) or DWT.^19^ CWT is a continuous representation that is well suited for transient spectral content, yet is highly redundant and difficult to extract quantitative features from. In contrast, the compact and orthogonal decomposition with time–frequency partitions of DWT make it better suited for reproducible biomarker derivation. Building on our previous mpIRD work, our primary wavelet analysis initially utilized the sym2 mother wavelet, which had demonstrated the highest energy-to-entropy ratio in the region of suspected macular cone response (13–27 Hz; detail decomposition level 6 [D6]) while preserving canonical PERG morphology.^38^ To complement this analysis, we also analyzed the Haar mother wavelet, as it uniquely exhibits the shortest filter length and linear phase response. Both of these facets enable high temporal precision and higher levels of decomposition that other mother wavelets are not capable of attaining.

For both wavelet families (sym2 and Haar), decomposition was performed with the periodic extension mode (“per”), yielding approximation (A) and detail (D) coefficients up to level 6 (A1–A6 and D1–D6, respectively) for sym2, and up to level 7 (A7 and D1–D7) for Haar. Because hardware bandpass filtering is 1 to 100 Hz, decomposition levels D1 to D3 (106–825 Hz) were discarded. Also, all but the last approximation level were removed as they contained redundant information with retained detail levels. Energies for each retained coefficient were computed as the square of each coefficient. To visualize the contribution of indices, partial reconstructions were generated by setting all wavelet indices to zero (except those of interest) and then applying the inverse DWT. The resulting reconstruction represents the portion of the original PERG captured within that indices time–frequency bands. Additional details regarding DWT coefficient/energy calculations and reconstruction can be found in our previous work.^19^^,^^38^

Five coefficients (sym2–D5-2, sym2–D6-2, sym2–D6-3, sym2–A6-3, and sym2–A6-4) previously demonstrated reconstruction of canonical N35, P50, and N95 peaks; their associated temporal ranges, frequency bands, and canonical marker can be found in Table 1^2^.^38^ We also assessed the mean of sym2–D6-2 with sym2–A6-3, mean(D6-2, A6-3), and the mean of sym2–D6-3 with sym2–A6-4, mean(D6-3, A6-4). When establishing a complementary representation of the PERG waveform with the Haar wavelet, we identified 10 indices with the highest mean energy across all HV subjects; only indices that temporally aligned with pertinent biological responses were retained. For this, we re-used the mpIRD dataset from our prior study (224 recordings from 56 subjects) to compare energy contributions across HV, OND, and mpIRD cohorts; full demographic details for this group can be found in Supplementary Table S1, as well as our previous work.^38^

**Table 1.** Time–Frequency Localization of the Five Key Symlet-2 Coefficients and Their Putative Physiological Generators

Index	Temporal Range (ms)	Frequency Band Range (Hz)	Associated Canonical Marker
D5-2	17–38	27–53	N35
D6-2	38–75	13–27	P50
D6-3	75–112	13–27	P50, N95
A6-3	75–112	0–13	P50, N95
A6-4	112–150	0–13	N95

**Table 2.** Demographic Profile of Analyzed HV and OND Cohort

				VA (logMAR), Mean ± SD	
Diagnosis	Subjects (Male; Female), *n*	Recordings, *n*	Age (Y), Mean ± SD	RE	LE
Healthy volunteers	67 (23; 44)	262	27.3 ± 17.7	0.20 ± 0.41	0.22 ± 0.42
Inherited optic atrophy	10 (6; 4)	34	27.0 ± 17.4	0.58 ± 0.88	0.73 ± 0.86
Optic neuropathy	3 (1; 2)	14	30.0 ± 25.2	0.63 ± 0.78	1.03 ± 0.64
Bilateral optic nerve atrophy	2 (2; 0)	10	35.5 ± 23.3	0.38 ± 0.23	0.21 ± 0.16

### 7N Extraction

To reproduce the 7N metric (equivalent to Daubechies 8 [db8]–D7-3) proposed by Hassankarimi et al.,^36^ all PERG recordings were resampled from the native 1700 Hz to 2839 Hz, zero-padded up to 512 samples, and min-max normalized to ±2 µV, yielding waveforms identical in structure to those of the original study. A seven-level (D1–D7) discrete wavelet transform with a db8 mother wavelet was applied. Detail energy indices at level 7 (D7-1, D7-2, D7-3, and D7-4) were isolated, and the percentage energy of the third index (D7-3) was computed as:

$$\begin{array}{c} 7N=\frac{db8-D7-3^{2}}{\sum_{i=1}^{4}db8-D7-i^{2}}\times 100 \end{array}$$

This value was stored as 7N and analyzed alongside the sym2 descriptors.

### Statistical Analysis

All statistics were performed in MATLAB R2024a. Between-group differences were quantified using the Mann–Whitney rank-biserial correlation |*r_rb_*|. Paired nonparametric comparisons between |N95–P50| and the top-performing RGC-specific wavelet index were performed using the Wilcoxon signed-rank test. To account for unit differences between amplitude (µV) and wavelet energy (µV^2^), *z*-scored values were compared. Standardizing features to *z*-scores removes unit differences and places all features on a common scale, enabling direct comparison across metrics. Test–retest reliability across multiple visits was quantified with the two-way mixed, single-measure intraclass correlation coefficient, ICC(2,1), computed on *z*-scores. Interpretation of ICC(2,1) was as follows: 0 to 0.5, poor; 0.5 to 0.75, moderate; 0.75 to 0.9, good; and 0.9 to 1.0, excellent.^42^ To compare wavelet features against PERG measures, we performed stratified subject-level bootstrap resampling (1000 iterations with a fixed random seed), sampling with replacement. For each resample, we computed |*r_rb_*| for each feature and quantified improvement relative to |P50–N35| and |N95–P50| as Δ|*r_rb_*|. One-sided bootstrap *P* values (*P_boot_*) were defined as the proportion of resamples in which Δ|*r_rb_*| ≤ 0.

## Results

### Validation of Macular-Cone Wavelet Marker sym2–D6-2

**Figure 2.**
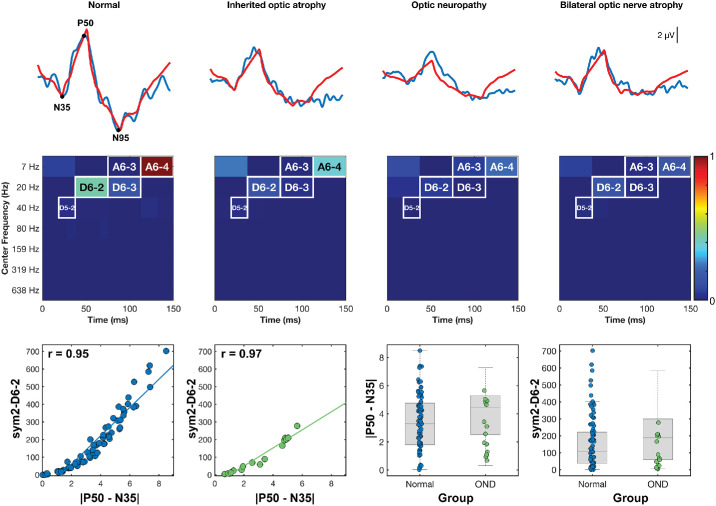
**Validation of the macular cone wavelet biomarker, sym2–D6-2.** (*Top*) PERG waveform (*blue*) from one HV eye and three OND phenotypes, with reconstructions from the five key sym2 indices plotted (*red*). (*Middle*) Associated scalograms locate the five indices in the time–frequency space. (*Bottom left*) The sym2–D6-2 energy correlated nearly perfectly with the traditional cone metric |P50–N35| in HV subjects (*r_corr_* = 0.95) and in the independent OND cohort (*r_corr_* = 0.97). (*Bottom right*) Tukey boxplots show substantial overlap between groups for |P50–N35| and sym2–D6-2, indicating that function is preserved in OND and that sym2–D6-2 is measuring the same metrics. Boxplots illustrate overlapping distributions between HV and OND for both |P50–N35| and sym2–D6-2, consistent with preserved function in OND.

To first confirm that our previously identified feature sym2–D6-2 does indeed represent macular cone responses as obtained by |P50–N35|, we examined it in an independent OND cohort. Figure 2 (top row) shows original PERG traces from a HV and three OND phenotypes (inherited optic atrophy, optic neuropathy, and bilateral optic nerve atrophy) with five-coefficient signal reconstructions overlaid in red. In each OND example, the P50 peak is largely preserved while the N95 trough is reduced, consistent with the preferential RGC loss of OND. Although P50 voltage and sym2–D6-2 energy for the HV recording were higher than that of the associated OND recordings, these parameters are relatively preserved, given the subjects pathology. In contrast, indices D6-3 (75–112 ms, 13–27 Hz) and A6-4 (0–13 Hz, 112–150 ms) demonstrated a far greater decrease in energy across OND recordings compared to the HVs. This pattern visually reinforces the specificity of D6-2 for cone-driven P50 activity.

Quantitatively, sym2–D6-2 energy is strongly correlated with the canonical |P50–N35| amplitude in both the HV group (*r_corr_* = 0.95) and the OND group (*r_corr_* = 0.97) (Fig. 2, bottom row). Table 3 shows that the mean energy of D6-2 was 46% lower in the OND than in HVs (100.73 ± 93.01 µV^2^ vs. 185.36 ± 168.59 µV^2^), whereas |P50–N35| showed only a 15% reduction (4.23 ± 1.77 µV vs. 5.00 ± 2.42 µV). This greater proportional drop in D6-2 suggests that the wavelet feature may be more sensitive than raw amplitude to subtle macular cone attenuation. Test–retest reliability was also high, ICC(2,1) = 0.88, matching the stability of |P50–N35|. Together, these results highlight that sym2–D6-2 effectively captures P50-driven cone activity, as well as serving as a sensitive metric for detecting macular function, or lack thereof.

**Table 3.** Group Means, Diagnostic Performance, and Effect Sizes of Traditional Temporal Measures Versus Symlet-2 Wavelet-Derived Biomarkers

Feature	Time (ms)	Frequency (Hz)	HV Energy (µV^2^), Mean ± SD	OND Energy (µV^2^), Mean ± SD
|P50–N35|	∼50	—	5.00 ± 2.42	4.23 ± 1.77
|N95–P50|	∼95	—	5.70 ± 2.53	4.18 ± 1.60
sym2–D5-2^a^	19–38	27–53	8.16 ± 9.21	14.27 ± 12.54
sym2–D6-2	38–75	13–27	185.36 ± 168.59	100.73 ± 93.01
sym2–A6-3	75–112	0–13	146.80 ± 183.95	72.45 ± 69.06
mean(D6-2, A6-3)	38–112	0–27	166.08 ± 150.62	86.59 ± 63.90
sym2–D6-3	75–112	13–27	47.18 ± 47.57	20.98 ± 24.26
sym2–A6-4	112–150	0–13	132.86 ± 109.08	47.14 ± 61.95
mean(D6-3, A6-4)	75–150	0–27	90.02 ± 76.04	34.06 ± 42.05
7N (db8–D7-3)	90–135	11–22	12.82 ± 10.61	13.46 ± 9.23

a The LE recordings of ID 78 and the RE recordings of ID 261 were not included in the analysis as their sym2–D5-2 values were >4 SD higher than the mean of the remaining values.

### Evaluation of RGC-Sensitive Wavelet Biomarkers

Based on their temporal and frequency localization to the |N95–P50| region (Table 1), three sym2 features, D6-3, A6-4, and their combined mean(D6-3, A6-4), were evaluated as candidate RGC biomarkers. All three demonstrated large mean energy reductions in OND compared to HVs: 56%, 65%, and 62% for D6-3, A6-4, and mean(D6-3, A6-4), respectively (Table 3). These values indicate a greater relative loss in the wavelet-derived metrics than conventional |N95 – P50| amplitude, potentially suggesting a greater sensitivity for RGC dysfunction.

**Figure 3.**
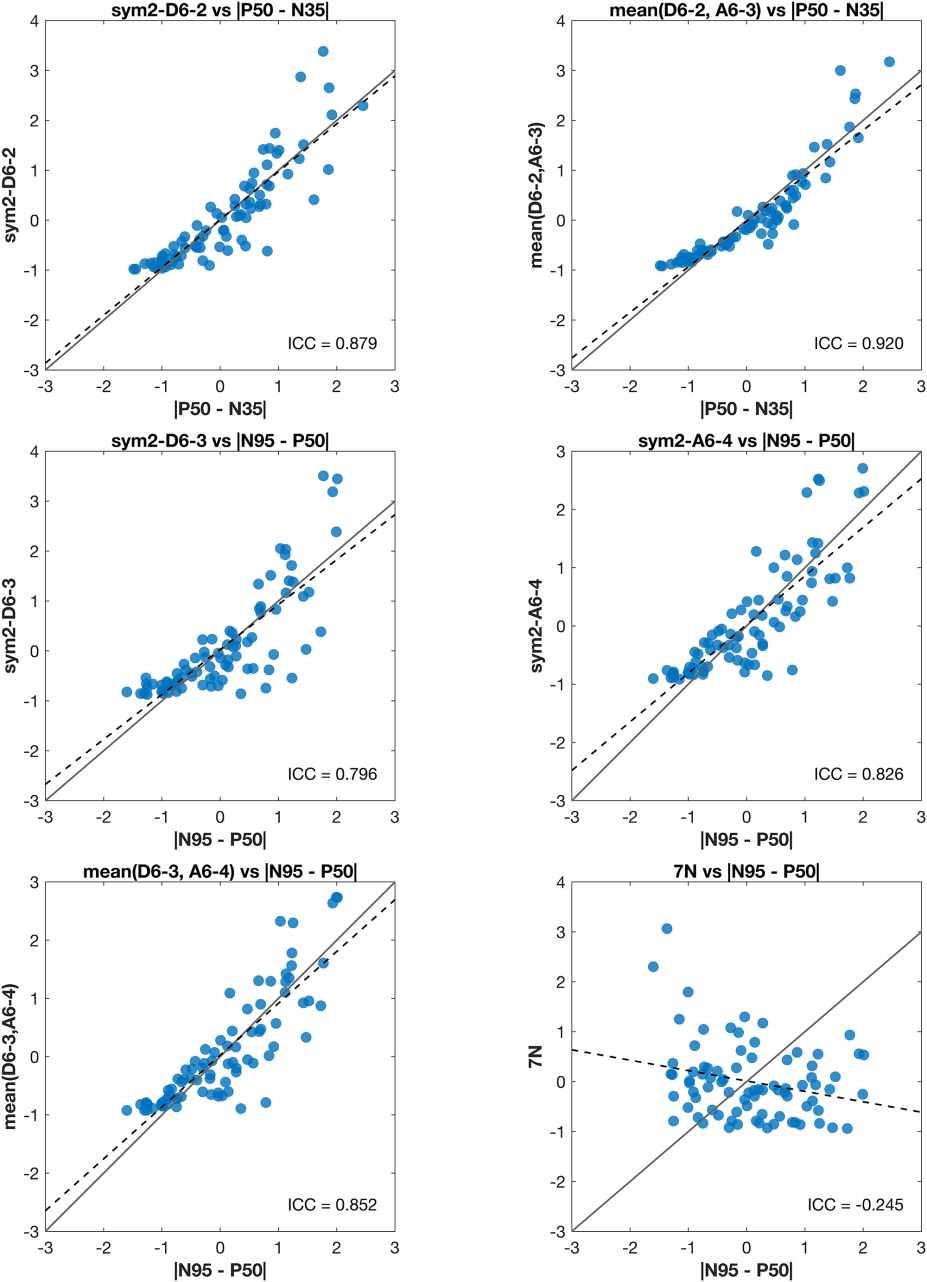
**Concordance and reliability of wavelet features versus conventional amplitude metrics.** The *z*-scored (no unit) scatterplots compare each wavelet index with its time-domain analog (*n* = 82 recordings; note that recordings from subject ID 78 were not included in this analysis as these were almost entirely large-amplitude sinusoids that presented as a single large outlier). *Solid line* indicates identity; *dashed line*, Deming regression.

In terms of discrimination, Table 3 shows that sym2–A6-4 achieved the highest effect size (|*r_rb_*| = 0.549) of the three, outperforming |N95–P50| (|*r_rb_*| = 0.358). The combined metric mean(D6-3, A6-4) also performed well (|*r_rb_*| = 0.511). Both A6-4 and the combined metric were strongly correlated with |N95 – P50| (*r_corr_* = 0.91 and *r_corr_* = 0.93, respectively), underscoring their ability to capture RGC response. Test–retest reliability was good for all three metrics—ICC(2,1) = 0.80, 0.83, and 0.85 for D6-3, A6-4, and mean(D6-3, A6-4), respectively—confirming measurement stability across subjects (Fig. 3). Bootstrap benchmarking against PERG measures (Table 4) showed that sym2–A6-4 yielded a statistically reliable improvement in rank-biserial separation relative to both |P50–N35| and |N95–P50| (Δ|*r_rb_*| = 0.362 and 0.187; *P_boot_* = 0.005 and 0.036, respectively). Mean(D6-3, A6-4) also improved with |P50–N35| (Δ|*r_rb_*| = 0.325; *P_boot_* = 0.011), whereas 7N did not outperform either.

**Table 4.** Bootstrap-Estimated Improvement in Rank-Biserial Separation of OND Relative to PERG Measures

Feature	|*r_rb_*|	Δ|*r_rb_*| vs. |P50–N35| (95% Bootstrap CIs)	*P_boot_*, Δ|*r_rb_*| ≤ 0 vs. |P50–N35|	Δ|*r_rb_*| vs. |N95–P50| (95% Bootstrap CIs)	*P_boot_*, Δ|*r_rb_*| ≤ 0 vs. |N95–P50|
sym2–D5-2	0.300	0.118 (−0.378, 0.534)	0.309	−0.058 (−0.545, 0.461)	0.597
sym2–D6-2	0.299	0.116 (−0.070, 0.284)	0.094	−0.060 (−0.186, 0.082)	0.825
sym2–A6-3	0.271	0.094 (−0.120, 0.321)	0.218	−0.082 (−0.334, 0.201)	0.730
Mean (D6-2, A6-3)	0.319	0.138 (−0.373, 0.252)	0.035	−0.038 (−0.169, 0.088)	0.714
sym2–D6-3	0.353	0.168 (−0.110, 0.435)	0.101	−0.008 (−0.204, 0.207)	0.559
sym2–A6-4	0.549	0.362 (0.085, 0.632)	0.005	0.187 (−0.018, 0.416)	0.036
Mean (D6-3, A6-4)	0.511	0.325 (0.045, 0.600)	0.011	0.150 (−0.043, 0.360)	0.077
7N (db8-D7-3)	0.084	−0.039 (−0.381, 0.277)	0.575	−0.214 (−0.528, 0.147)	0.89

|*r_rb_*| indicates the magnitude of Mann–Whitney rank-biserial separation between HV and OND. Δ|*r_rb_*| denotes bootstrap-estimated improvement relative to the indicated PERG measure. One-sided bootstrap *P* values (*P_boot_*) represent the proportion of resamples with Δ|*r_rb_*| ≤ 0 across 1000 subject-level resamples. CI, confidence interval.

**Figure 4.**
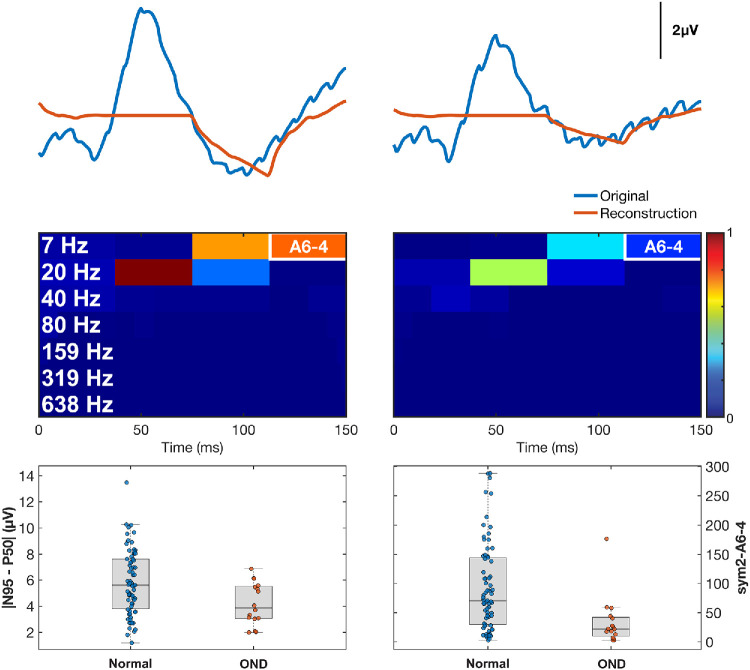
**Sym2–A6-4 captures RGC dysfunction better than |N95–P50|.** (*Top*) Averaged HV (*left*) and averaged OND (*right*) recordings (*blue*) and their associated sym2–A6-4 reconstructions (*red*). (*Middle*) Scalograms highlight the A6-4 window (112–150 ms, 0–13 Hz). (*Bottom*) Boxplots show a considerable overlap in the |N95–P50| metric, but sym2–A6-4 demonstrated a pronounced reduction of energy and minimal overlap. These results suggest that sym2–A6-4 is a sensitive, physiologically plausible marker of ganglion cell/optic nerve impairment.

Figure 4 (top row) compares mean PERG traces of HVs (left) and OND subjects (right). The red reconstructions from the sym2–A6-4 index closely follow the original traces in the later portion of the waveform (around the N95), but are notably attenuated in OND, reflecting loss of RGC-driven activity. In the middle row, the group-averaged scalograms show that the A6-4 time–frequency window (112–150 ms, 0–13 Hz) had high energy in the HVs but markedly reduced energy in the OND group. This indicates that A6-4 isolates a late, low-frequency component of the PERG that is preferentially diminished in OND. The box plots in the bottom row demonstrate this visually, as the |N95–P50| values for the two groups considerably overlap, whereas the A6-4 energy distributions do not as much, supporting the latter as a potentially sensitive marker of RGC dysfunction. Paired nonparametric testing (Wilcoxon signed-rank) was performed between *z*-scored |N95–P50| and sym2–A6-4 values to account for unit differences. No significant difference was found (*n* = 83; *P* = 0.69; *r_rb_* = −0.05), indicating that the two metrics have comparable central tendencies when standardized.

### Complementary Haar Wavelet Representation of PERG Signals

Although the sym2-based features effectively captured both macular cone and RGC dysfunction, the Symlet family has a longer filter length, limiting temporal precision and extent of decomposition. To address this limitation, we explored the Haar wavelet, as it has the shortest possible filter length and a linear phase response, both of which enable sharp temporal localization and higher levels of decomposition.

**Figure 5.**
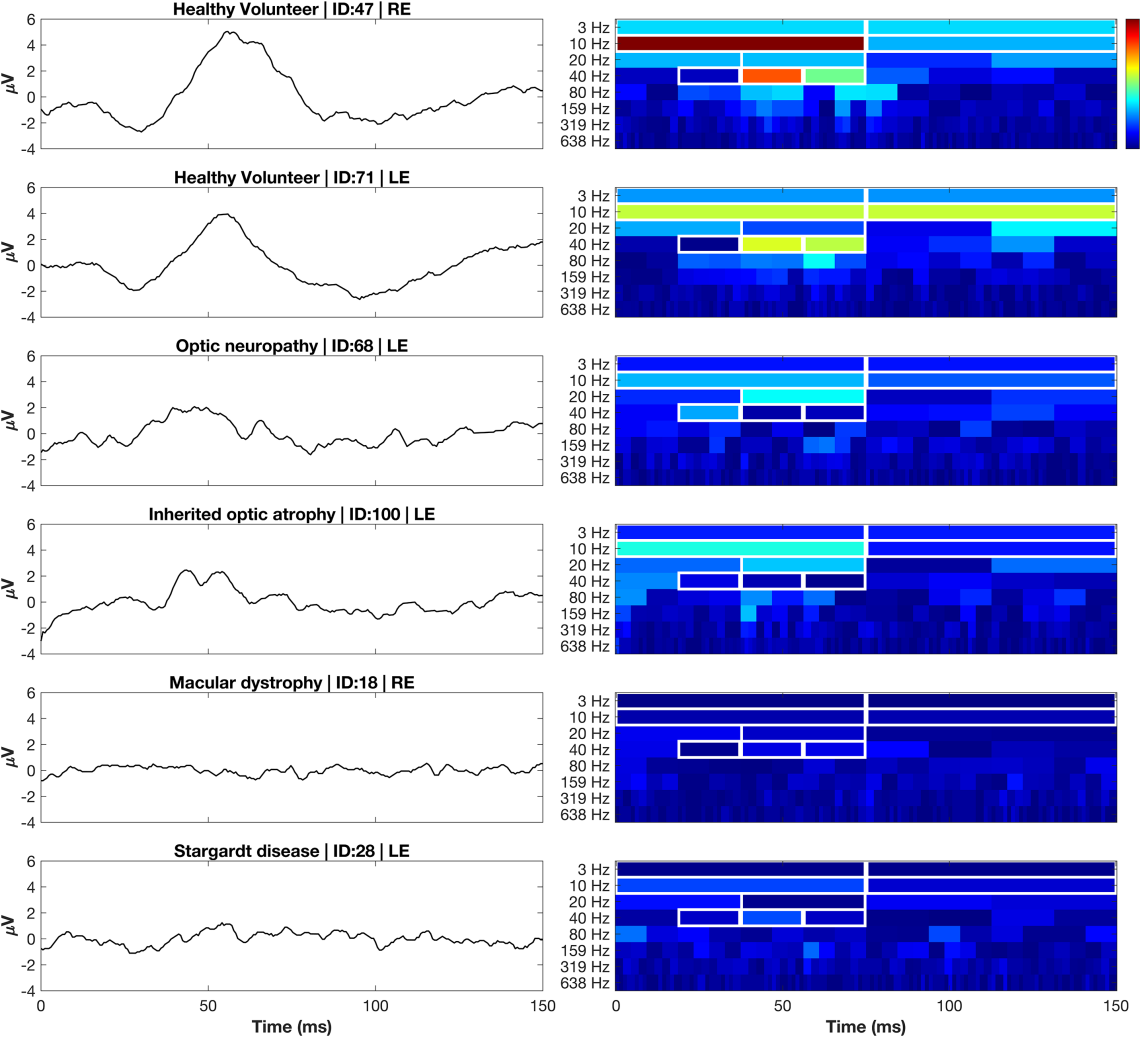
**Representative PERG waveforms (*left*) and Haar scalograms (*right*) from healthy and diseased eyes.** Each row pairs a trace (µV, *y*‑axis) with its time–frequency energy map (percent of total signal energy from 0%–100%; *color bar*). Two HV recordings were followed by four disease phenotypes spanning OND and mpIRD: optic neuropathy, inherited optic atrophy, macular dystrophy, and Stargardt disease. *White rectangles* outline the eight retained Haar indices: A7‑1/2 (0–7 Hz, 0–75 ms and 75–150 ms), D7‑1/2 (7–13 Hz, 0–75 and 75–150 ms), D6‑2 (13–27 Hz, 38–75 ms), and D5‑2/3/4 (27–53 Hz, 19–75 ms), which together captured ∼80 % of PERG energy in HV eyes. HV eyes show concentrated low‑frequency energy aligned with canonical P50 and N95 responses, whereas OND and mpIRDs exhibit marked attenuation of energy within these indices.

Haar wavelet analysis identified several indices capturing substantial energies while temporally aligned with physiologically relevant PERG features. The highest energy indices in HV subjects included Haar–D7-1 (0–75 ms; 7–13 Hz; 258.09 ± 268.91 µV^2^), Haar–D7-2 (75–150 ms; 7–13 Hz; 127.36 ± 132.52 µV^2^), and Haar–D6-2 (38–75 ms; 13–27 Hz; 42.41 ± 57.86 µV^2^). Low-frequency responses were also well captured by Haar–A7-1 (0–75 ms; 0–7 Hz; 38.80 ± 30.44 µV^2^) and Haar–A7-2 (75–150; 0–7 Hz; 37.24 ± 29.41 µV^2^). Exemplar HV, OND, and mpIRD recordings and their associated Haar scalograms representing these selected indices are outlined in Figure 5. A full outline of the retained indices is available in Supplementary Table S2.

Compared to the sym2 decomposition, the Haar representation yielded a greater number of physiologically relevant indices that collectively cover more specific and narrower time–frequency regions. For example, within the ∼40-Hz band in Figure 5, Haar analysis identified three discrete indices spanning 18.75 to 75 ms, whereas the sym2 decomposition represented this same frequency band with a single index (D5-2). This finer subdivision may improve both sensitivity and specificity by isolating physiologic generators into distinct time–frequency bins. Moreover, because the Haar transform exhibits zero phase distortion, these indices preserve true temporal alignment of the underlying bioelectric events, enhancing interpretability.

### Independent Evaluation of the Glaucoma-Derived 7N Metric

The 7N metric originally proposed for delineating early primary open-angle glaucoma (POAG) from HVs showed no meaningful ability to delineate HV recordings from OND, as outlined in Table 3. Mean 7N energy on average differed by only 5.0% between the OND and HV groups, with a low effect size (|*r_rb_*| = 0.084). Test–retest reliability was poor, ICC(2,1) = −0.25, as shown in Figure 3.

## Discussion

This is the second study, to our knowledge, to evaluate the PERG–IOBA dataset and the first to directly assess the RGC response of these recordings. This study reinforces the utility of DWT analysis for deriving sensitive and robust time–frequency measures from PERG recordings, with select features demonstrating superior performance over traditional peak-selection methods. We validated the previously established macular cone index sym2–D6-2 with an independent OND cohort. Similarly, we evaluated three novel RGC descriptors: sym2–D6-3, sym2–A6-4, and their mean, mean(D6-3, A6-4). Of these three metrics, sym2–A6-4 yielded the highest effect size compared to the conventional |N95–P50| measure. Together, these five indices provide a comprehensive time–frequency framework capable of assessing both outer and inner retinal integrity. Additionally, we established a compact set of Haar indices that capture a majority of the physiological responses of PERG. This collection of Haar indices offers a temporally faithful alternative to Symlet-based analysis, as well as a higher decomposition level for finer temporal and spectral delineation.

In a previous study of ours, the energy index sym2–D6-2 (38–75 ms, 13–27 Hz) yielded the largest effect size (|*r_rb_*| = 0.64) between HVs and those with mpIRD. Validation in our curated OND cohort as shown in Figure 2 demonstrated a Pearson correlation of *r_corr_* = 0.97 for |P50–N35| versus sym2–D6-2, as well as overlapping Tukey boxplots. OND demonstrated selective RGC and optic nerve loss with largely preserved macular cone function. Although studies have demonstrated retrograde loss of macular cone photoreceptors due to RGC loss in such conditions,^43^ there is still an appreciable macular cone response that can still be useful for assessing the ability of this metric to capture macular cone response faithfully. Furthermore, Figure 2 represents one HV and three different OND pathologies, with the latter three demonstrating moderately preserved macular cone responses. Although these results are promising, they are derived retrospectively from a single site; prospective validation with an external dataset of HVs is necessary to confirm these findings.

With this same OND cohort, we attempted to identify time–frequency indices that largely differed between this group and HVs using effect sizes. It should be noted that these indices target the latter half of the PERG waveform dominated by RGC contributions, which we expected to be characteristically attenuated in our OND group. Sym2-A6-4 (112–150 ms, 0–13 Hz) achieved the largest effect size (|*r_rb_*| = 0.549) and was the only one to yield a significant *P_boot_* when comparing effect sizes with |N95 – P50|. The combined metric, mean(D6-3, A6-4), produced a slightly lower effect size (|*r_rb_*| = 0.511) but demonstrated superior test–retest reliability, ICC(2,1) = 0.85 versus 0.83. Although sym2–A6-4 offers the better separation, mean(D6-3, A6-4) remains consistently linked to the traditional |N95 – P50| measure, as demonstrated in our ICC analysis and in Pearson correlations (*r_corr_* = 0.93 vs. 0.91). A similar finding can be noted in sym2–D6-2 and mean(D6-2, A6-3), ICC(2,1) = 0.88 versus 0.92. Mean(D6-2, A6-3) was the only metric to achieve excellent reliability. Although individual metrics such as sym2–D6-2 and sym2–A6-4 produced greater effect sizes individually, the combined metrics, such as mean(D6-3, A6-4) and mean(D6-2, A6-3), demonstrated stronger correlations and higher reliability with the traditional amplitude-based measures (|N95 – P50| and |P50–N35|, respectively). These combined metrics may offer an optimal compromise of improved performance while also capturing similar information, as they, too, encompass a wide temporal range of the signal and multiple frequency bands.

Nonetheless, we are particularly keen on resolving the exact physiological response sym2–A6-4 is capturing and believe it may be optic nerve spiking in response to the checkerboard reversal of the standard PERG. Previous studies by Luo and Frishman^44^ have demonstrated that the pattern reversal rate of the PERG results in a direct optic nerve action potential, with a pattern reversal of 4 rps yielding ∼4 optic nerve spikes per second. Given that sym2–A6-4 occurred in the latter half of the PERG signal (75–150 ms), demonstrated large differences between HVs and subjects with OND, and encompassed the spectral range of 0 to 13 Hz, it is plausible that this metric captures the response of the optic nerve to the 4-rps stimulus. To further evaluate this, we selectively assessed Haar–A7-2 (75–150 ms, 0–7 Hz), which represents a frequency resolution twice as fine as sym2–A6-4 (0–13 Hz). We found the mean ± SD in HVs and subjects with OND to be 37.24 ± 29.41 µV^2^ and 13.91 ± 12.81 µV^2^, respectively. This can be appreciated in Figure 5, with HV subjects Haar–A7-2 exhibiting higher energies than those of the following four diseased recordings. Although the effect size of this measure is slightly lower (|*r_rb_*| = 0.500) than sym2–A6-4 (|*r_rb_*| = 0.549), it is likely due to the sym2 wavelet being structurally similar to the waveform it is reconstructing, whereas Haar transformations are purely orthogonal and especially effective for sharp, abrupt transitions.

We then extended analysis of the Haar wavelet to obtain a set of physiologically representative indices in HV recordings based on highest mean energies. In HV subjects, these eight selected indices (outlined in Supplementary Table S2) accounted for a mean 80% ± 13% of the total PERG energy, indicating that most of the signal resides in these time–frequency indices. Similarly, a high proportion is preserved in the OND cohort with a mean energy across all subjects of 78% ± 11%. This was only not the case for mpIRD traces, which showed these indices retaining 62% ± 16% of energy across respective recordings. The lower percentage likely reflects the fact that mpIRDs exhibit global reduction in early cone and later RGC responses. Thus, as signal amplitude decreases in physiologically representative indices, energy in physiologically irrelevant indices comprise a larger portion of the signal. This can be visually appreciated in Figure 5, where it can be seen that mpIRD scalograms presented with sparse energies across all levels, resulting in higher frequency indices contributing relatively more to the total signal energy.

A previous study by Hassankarimi et al.^36^ is, to our knowledge, the only report to apply DWT analysis to PERG recordings in subjects with POAG, isolating the 7N metric (90–135 ms, 11–22 Hz) using the Daubechies 8 mother wavelet. This metric ultimately performed poorly on all accounts, as shown in Tables 3 and 4 and Figure 3 (|*r_rb_*| = 0.084), ICC(2,1) = −0.25. POAG and OND both culminate in RGC dysfunction, yet their pathogenic processes differ. POAG is a progressive optic neuropathy driven by biomechanical, vascular, and neuro-inflammatory insults injuring axons, with somatic loss occurring months to years later. Conversely, ONDs such as optic atrophy 1 (OPA1)- or mitofusion 2 (MFN2)-related optic neuropathies arise from primary mitochondrial or axoplasmic defects that cause early, diffuse somatic stress and can spare distal axons until late stages. These divergent spatiotemporal patterns may alter RGC failure pathogenesis expressed in the time–frequency domain and explain poor performance in feature validation. Poor performance due to methodological transformations is less likely to be the cause when using DWT. Resampling steps may alter spectra introducing high-frequency artifacts, and zero padding may introduce edge artifacts. However, the 7N feature is centered mid-epoch away from where edge artifacts may arise and is localized in the 11- to 22-Hz frequency bin, avoiding high-frequency artifacts from interpolation. Nonetheless, assessment of this index with a separate set of POAG recordings, with the same recording protocol, is necessary to further evaluate this claim.

A limitation of the present study is that localization is fixed by dyadic partitioning, such that temporal delays or shifts in physiological response result in energy spreading across adjacent indices. Alternative approaches, including local wavelet maximum methods by Gauvin et al.,^23^ address this limitation by using an adaptive sliding window. However, this is at the cost of invertibility, orthogonality, and substantial redundancy. In this context, an important next step would be to determine whether time–frequency biomarkers provide incremental information beyond a joint model of conventional amplitudes and implicit times, rather than considering each in isolation. One other consideration is that OND severity and stage are not provided in the PERG–IOBA dataset; thus, we are unable to determine whether our OND cohort spans subtle preclinical dysfunction or advanced optic atrophy. As a result, the present classification partially reflects separation of overt disease and prospective validation in clinically graded OND. Ideally, other clinical measures such as retinal nerve fiber layer thickness and genotype are available to contextualize functional results.

Although the present study utilizes the only public PERG dataset containing both HV and pathological recordings, its retrospective single-center design inevitably limits generalizability. Furthermore, there may be subtle differences in acquisition parameters between this and other studies; wavelet features tuned specifically to the 1700-Hz sampling rate and 150-ms epoch used in this study may shift when higher sampling rates or longer epochs are employed. Similarly, our OND pathologies are non-descriptive in that we only know they have an OND. It would be interesting to see how these time–frequency metrics measure with genotype–phenotype information. Thus, future studies should consider the following:

• Pursue a multicenter, prospective validation using harmonized acquisition settings.
• Alter recording protocol (e.g., sampling rate of 3,413,33 Hz for 150-ms signal) to obtain a signal that would enable a higher level of decomposition—a signal of 512 samples. This would permit finer spectral binning and assessment of higher order wavelets.
• Assess detail decomposition level D4, as this level potentially contains biologically relevant information. However, due to the lower energies, this metric was not retained. A dynamic approach that evaluates the maximum or mean of several indices, similar to what was done by Gauvin and colleagues,^23^ would be appropriate.
• Given that the cellular contributions of the PERG dynamically adjust in response to checker size, a valuable next step would be to assess how both temporal measures and these identified energy indices evolve in response to changes in check size, confirming whether or not these measures are capturing the same response.^23^
• Investigate longitudinal measures that assess wavelet energies to traditional PERG metrics, as well as structural OCT and visual field trajectories.
• Evaluate how wavelet-derived metrics associate with specific genotype-phenotype profiles.
• Integrate machine-learning classifiers,^45^ with semantic ontologies to enhance disease classification,^46^ as well as incorporating genotype–phenotype insights driven by artificial intelligence diagnostic frameworks.^47^

## Conclusions

Applying DWT to PERG in an OND cohort yields a multidimensional quantitative framework that validates a previously identified macular cone metric (sym2–D6-2), as well as establishing measures of RGC response—sym2–D6-3, sym2–A6-4, and mean(D6-3, A6-4)—that outperform the traditional temporal metrics |P50–N35| and |N95–P50|. We also developed a compact Haar time–frequency representation, selecting eight indices that on average capture about 80% of the PERG signal, offering a phase-shift–free alternative to Symlet-based features. Finally, we have provided the first external validation of the DWT 7N metric with our OND cohort.

## Supplementary Material

Supplement 1
