# Supplementary material for: Wavelet-Based Pattern ERG Biomarkers Outperform Temporal Amplitude Measures for Functional Stratification in Optic Nerve Disease
Source: Transl Vis Sci Technol. 2026 Mar 11;15(3):13. doi: 10.1167/tvst.15.3.13 (PMC12988678; doi:10.1167/tvst.15.3.13)
Supplement: Supplement 1 [file tvst-15-3-13_s001.docx]

| **Supplementary Table 1. mpIRD cohort overview by diagnostic group.** | | | | |
| --- | --- | --- | --- | --- |
| Diagnosis | # of Subjects (Male/Female) | Recordings | Age (years, mean ± SD) | VA (logMAR, mean ± SD; RE / LE) |
| Cone-Rod Dystrophy | 12 (5/7) | 46 | 32.2 ± 15.9 | 0.61 ± 0.45 / 0.55 ± 0.40 |
| Macular Dystrophy | 29 (13/16) | 114 | 41.0 ± 17.1 | 0.41 ± 0.37 / 0.40 ± 0.36 |
| Stargardt disease | 16 (6/9) | 64 | 36.2 ± 15.9 | 0.57 ± 0.77 / 0.57 ± 0.56 |
| mpIRD: Macular-predominant inherited retinal disease, SD: Standard deviation, VA: Visual acuity, RE: Right Eye, LE: Left Eye | | | | |

| **Supplementary Table 2. Retained Haar indices and their average energy profiles across Healthy Volunteer recordings** | | | |
| --- | --- | --- | --- |
| Feature | Energy  (μV^2^, mean ± SD) | Time Range (ms) | Frequency Band (Hz) |
| D7‑1 | 258.09 ± 268.91 | 0 -75 ms | 7-13 Hz |
| D7‑2 | 127.36 ± 132.52 | 75 -150 ms | 7-13 Hz |
| D6‑2 | 42.41 ±  57.86 | 38-75 ms | 13-27 Hz |
| A7‑1 | 38.80 ±  30.44 | 0-75 ms | 0-7 Hz |
| A7‑2 | 37.24 ±  29.41 | 75-150 ms | 0-7 Hz |
| D5‑3 | 33.42 ±  42.19 | 38- 56 ms | 27-53 Hz |
| D5‑4 | 28.67 ±  39.49 | 56-75 ms | 27-53 Hz |
| D5‑2 | 13.37 ±  55.97 | 19-37 ms | 27-53 Hz |

SD: Standard deviation
